# Impact of telemedicine on glycemic control in type 2 diabetes mellitus during the COVID-19 lockdown period

**DOI:** 10.3389/fendo.2023.1068018

**Published:** 2023-02-03

**Authors:** Abrar M. Al-Mutairi, Mohammad A. Alshabeeb, Salah Abohelaika, Fadhel A. Alomar, Keshore R. Bidasee

**Affiliations:** ^1^ Research Unit, College of Applied Medical Sciences, King Saud bin Abdulaziz University for Health Sciences (KSAU-HS), Riyadh, Saudi Arabia; ^2^ King Abdullah International Medical Research Center (KAIMRC), Riyadh, Saudi Arabia; ^3^ Ministry of National Guard Health Affairs (MNG-HA), Riyadh, Saudi Arabia; ^4^ King Saud bin Abdulaziz University for Health Sciences (KSAU-HS), Riyadh, Saudi Arabia; ^5^ Department of Clinical Pharmacology, Qatif Central Hospital, Qatif, Saudi Arabia; ^6^ Department of Pharmacology and Toxicology, College of Clinical Pharmacy, Imam Abdulrahman Bin Faisal University, Dammam, Saudi Arabia; ^7^ Departments of Pharmacology and Experimental Neuroscience, University of Nebraska Medical Center, Omaha, NE, United States; ^8^ Department of Environment and Occupational Health, University of Nebraska Medical Center, Omaha, NE, United States; ^9^ Nebraska Redox Biology Center, Lincoln, NE, United States

**Keywords:** telemedicine, diabetes mellitus, glycated hemoglobin A1c, COVID-19, lockdown

## Abstract

**Background:**

The lockdown at the start of coronavirus disease 2019 (COVID-19) pandemic in Saudi Arabia (March 2020 to June 2020) shifted routine in-person care for patients with type 2 diabetes mellitus (T2DM) to telemedicine. The aim of this study was to investigate the impact telemedicine had during this period on glycemic control (HbA1c) in patients with T2DM

**Methods:**

4,266 patients with T2DM were screened from five Ministry of National Guard Health Affairs hospitals in the Kingdom of Saudi Arabia. Age, gender, body mass index (BMI), HbA1c (before and after the COVID-19 lockdown), duration of T2DM, comorbidities and antidiabetic medications data were obtained. Mean and standard deviation of differences in HbA1c were calculated to assess the impact of telemedicine intervention. Correlations between clinically significant variances (when change in the level is ≥0.5%) in HbA1c with demographics and clinical characteristic data were determined using chi square test.

**Results:**

Most of the participants were Saudis (97.7%) with 59.7% female and 56.4% ≥60 years of age. Obesity was 63.8%, dyslipidemia 91%, and hypertension 70%. Mean HbA1c of all patients slightly rose from 8.52% ± 1.5% before lockdown to 8.68% ± 1.6% after lockdown. There were n=1,064 patients (24.9%) whose HbA1c decreased by ≥0.5%, n =1,574 patients whose HbA1c increased by ≥0.5% (36.9%), and n =1,628 patients whose HbA1c changed by <0.5% in either direction (38.2%). More males had significant improvements in glycemia compared to females (28.1% vs 22.8%, p<0.0001), as were individuals below the age of 60 years (28.1% vs 22.5%, p<0.0001). Hypertensive individuals were less likely than non-hypertensive to have glycemic improvement (23.7% vs 27.9%, p=0.015). More patients on sulfonylureas had improvements in HbA1c (42.3% vs 37.9%, p=0.032), whereas patients on insulin had higher HbA1c (62.7% vs 56.2%, p=0.001). HbA1c changes were independent of BMI, duration of disease, hyperlipidemia, heart and kidney diseases.

**Conclusion:**

Telemedicine was helpful in delivering care to T2DM patients during COVID-19 lockdown, with 63.1% of patients maintaining HbA1c and improving glycemia. More males than females showed improvements. However, the HbA1c levels in this cohort of patients pre- and post-lockdown were unsatisfactorily high, and may be due to in part lifestyle, age, education, and hypertension.

## Introduction

As a result of the global pandemic that emerged at the end of 2019 due to the severe acute respiratory syndrome coronavirus 2 (SARS-CoV-2) (1), many countries began closing their boarders and implemented stay-at home (lockdown) measures to limit the spread of the virus and protect their citizens (2). This lockdown inadvertently restricted patients with per-existing comorbidities to visit outpatient clinics, limited their physical activities, influenced their eating habits, and impacted their psychological status (3). These unprecedented conditions were expected to worsen glycemia in DM patients which highlights the importance of optimizing drug therapy in chronic and long-term DM patients. In addition, patients with DM require regular face-to-face communication with diabetic practitioners, and diabetic educators to achieve optimal glycemic control (4). This approach is needed to blunt multi-organ comorbidities that arise from the persistent hyperglycemia in DM patients (3–5).

Healthcare sectors in many countries adopted strategies to provide timely and effective care to DM patients during this period (6). In Brazil, providing educational materials on healthy habits, mental health, and diabetes management *via* telephone calls during the lockdown was an effective healthcare measure (7). Globally, use of telehealth increases from 1% to >50% of diabetes clinics during the pandemic (8). It also became possible to achieve tight glycemic control and prevent fluctuation in blood glucose levels with the recent advances in technology that allow virtual care (9). During the period 25^th^ March 2020 to 20^th^ June 2020 of the COVID-19 pandemic, the hospitals and health centers in the Saudi Arabia used telehealth to ensure patients receive the necessary health care and medications at timely manner. A recent study, that involved 270 Saudi patients on anticoagulants, confirmed that tele-pharmacy clinic was as effective as face-to-face consultations (10). Other studies also suggest that telehealth is useful in managing DM patients and improving glycated hemoglobin A1C levels (HbA1c) in DM patients (11, 12). A prospective cohort study conducted by Tourkmani et al. (2021) screened diabetic COVID-19 patients from Saudi Arabia and reported that telehealth had a significant positive impact on glycemic control, in high-risk diabetic patients with HbA1c levels decreasing from 9.98% to 8.32%. However, the number of diabetic patients in this study was relatively small (n=130) potentially limiting the conclusion (13). Here, we investigated the effect of telehealth on glycemic control in a larger number of T2DM (n=4,266) in various regions of Saudi Arabia during the COVID-19 pandemic lockdown.

## Methodology

The study was approved by the Institutional Review Board (IRB) at King Abdullah International Medical Research Centre (KAIMRC, NRC21R/463/11). This is a multicenter retrospective cohort study; the data was collected from the patient’s medical records at the Ministry of National Guard Health Affairs (MNGHA), Saudi Arabia. All information was obtained from the BestCare electronic health records (EHRs) system. The health system covers all MNGHA individuals, with free full-healthcare services in five hospitals in the three main regions (Central, Western, and Eastern) of Saudi Arabia.

**Figure 1:**
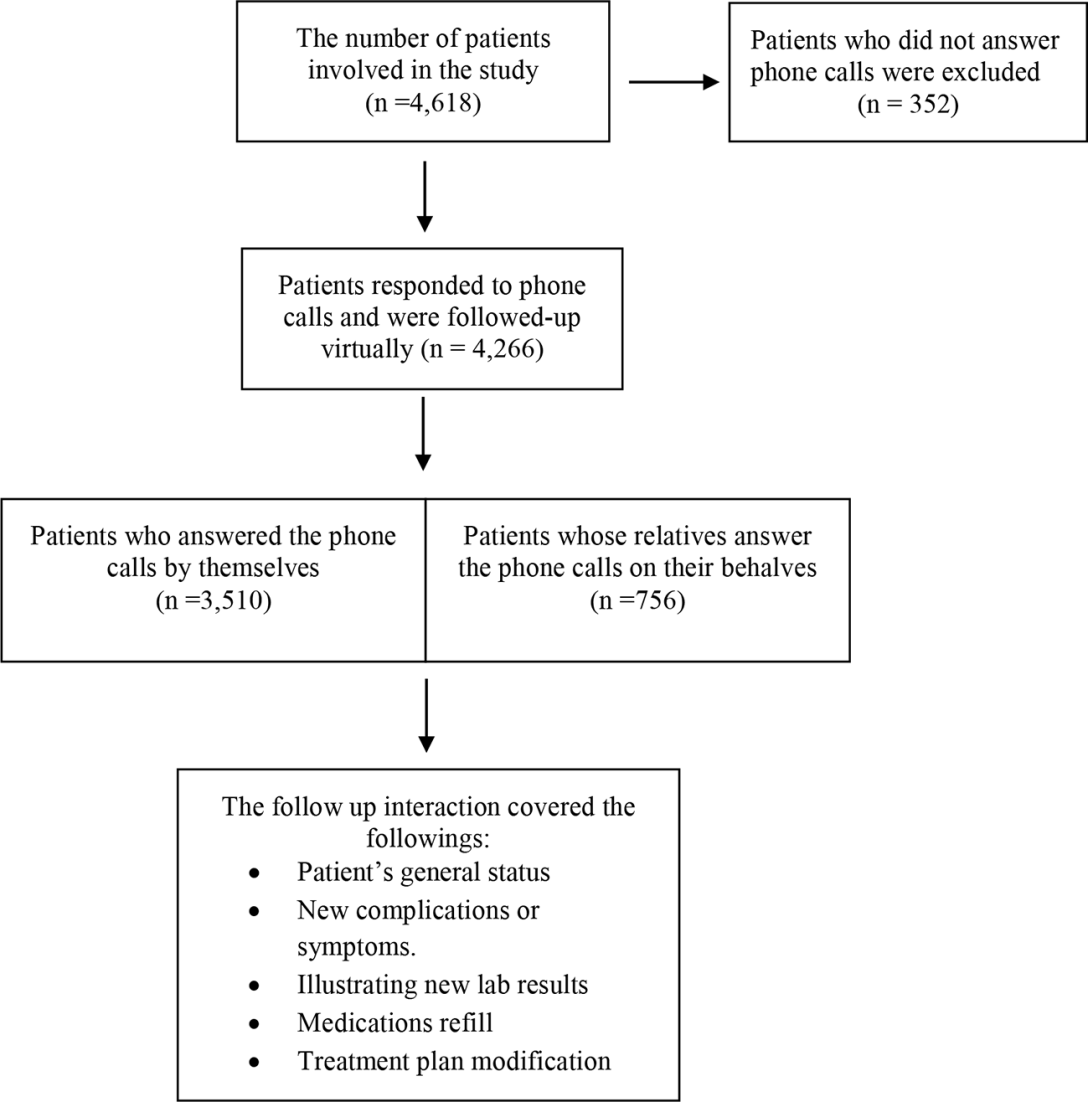
PRISMA flowchart illustrating the patients involved in virtual appointments.

The recruited patients in this study included (i) all adult patients with T2DM, (ii) had an HbA1c value of ≥6.5%, and (iii) answered the scheduled phone calls during the follow up period (25^th^ March 2020 to 20^th^ June 2020). Pre-diabetic patients (HbA1c level of 5.7%- 6.4%) and those who did not answer phone calls throughout the scheduled virtual clinics were excluded. As a result of these criteria, 4,266 patients were included in this study. **Figure 1** illustrates an inclusion and exclusion flowchart which also shows the main elements of focus during telemedicine communication and diabetic education. This involved a follow up of patient’s current status, documenting new episodes of complications or symptoms, reviewing recent laboratory results, ordering medications refill, and suggesting treatment plan modification when needed. The duration of T2DM, comorbidities, body mass index (BMI) classification, and prescribed medications were all recorded. The extracted data in particular HbA1c readings were grouped based on two primary time points: “pre-COVID-19 lockdown”, which included data from 15^th^ September 2019 until 24^th^ March 2020; and “post-COVID-19 lockdown”, which included data from 21^st^ June 2020 until 15^th^ September 2020.

Descriptive analyses are reported using mean (M) and standard deviation (SD) for continuous variables and frequencies and percentages for categorical variables. The HbA1c score changes between pre- and post-lockdown were calculated and the difference significance was assessed using the paired t-test. P-value ≤0.05 was used as a criterion to determine whether the observed differences are statistically significant. The American Diabetes Association suggests that changes in HbA1c mean levels below 0.5% are not clinically significant (14). The patients with clinically significant decreased HbA1c mean levels were compared to patients with clinically significant increased values using chi-square test to determine the impacting factors among demographic and clinical characteristics. Categorical covariates in the number and chemical classes of given drugs between the two compared groups were also assessed. The chosen statistical tests were run through using the Statistical Package for the Social Sciences (SPSS) software version 27.

## Result

A total of 4,618 T2DM patients were scheduled for the virtual integrated clinic at MNGHA facilities in Central, Western, and Eastern regions. Of these patients, 4,266 patients satisfied the inclusion criteria (**Figure 1**, with 2,547 females and 1,719 males (59.7% vs 40.3%, respectively). Baseline characteristics of the followed up patients are shown in **Table 1**. The majority of patients were Saudis (97.7%), with high percentage of them (82.5%) in the Central region. Patients aged ≥60 years represented the highest percentage of the participants (56.4%), and then those aged between 45–59 years old (36.8%). The younger patients ≤44 years old represented only 6.8%. Two thirds of the patients (66.3%) had diagnosed with T2DM for less than five years, and 63.8% were obese (BMI≥30). Most of the patients (95.2%) had at least one or more concomitant chronic illnesses; 3,029 (71.0%) patients had hypertension, 3,860 (90.5%) had hyperlipidemia, 348 (8.2%) had heart diseases, and 342 (8.0%) had kidney disease.

**Table 1:** Baseline demographic and clinical characteristics of recruited patients.

Characteristics		Number of patients followed-up virtually (n= 4,266)
**Gender**	Male	1,719 (40.3%)
	Female	2,547 (59.7%)
**Nationality**	Saudi	4,168 (97.7%)
	Non-Saudi	98 (2.3%)
**Regions**	Central	3,519 (82.5%)
	Western	439 (10.3%)
	Eastern	308 (7.2%)
**Age (years)**	18-44	291 (6.8%)
	45-59	1,568 (36.8%)
	≥60	2,405 (56.4%)
**BMI <18.5**	Underweight	10 (0.2%)
**18.5-24.9**	Healthy	296 (6.9%)
**25-29.9**	Overweight	1,206 (28.3%)
**≥30**	Obesity	2,720 (63.8%)
**Diabetes duration**	<5 years	2,829 (66.3%)
	>5 years	1,437 (33.7%)
Comorbidities		
Hypertension	Yes No	3,029 (71.0%) 1,237 (29.0%)
Hyperlipidemia	Yes No	3,860 (90.5%) 406 (9.5%)
Heart disease	Yes No	348 (8.2%) 3,918 (91.8%)
Kidney disease	Yes No	342 (8.0%) 3,924 (92.0%)

**Table 2** shows the mean difference before and after lockdown among the tested cohort; the overall mean HbA1c increased slightly from 8.52% ( ± 1.5%) before lockdown to 8.68% ( ± 1.6%) after lockdown. This change is not clinically significant (HbA1c level difference <0.5%). However, subgrouping of the study cohort indicated that one quarter of the patients (n=1,064, 24.9%) showed significant glycemic improvement (mean HbA1c decreased by 1.38%), versus 1,574 patients (36.9%) demonstrated poorer control (mean HbA1c increased by 1.36%), while 1,628 patients (38.2%) reported minimal non-significant HbA1c changes (<0.5%). Comparisons between these three groups were made to identify the demographic factors that are associated with these changes as shown in **Table 3**. Higher percentage of males was detected in the group of improved outcome than in females (28.1% vs 22.8%, respectively, p<0.0001). Moreover, higher improvement rates were identified among patients in Western region than in the Central and Eastern regions (29.6% vs 24.3 and 24.7%, respectively, p= 0.005). Also, percentage of patients aged <60 years was higher in the HbA1c improved group than in those aged ≥60 (28.1% vs 22.5%, respectively, p<0.0001). In addition, lower rates of improvement were noted in those with hypertension compared to non-hypertensive diabetic patients (23.7% vs 27.9%, p=0.015). There was no association between the changes in HbA1c and other variables including patients’ nationality, BMI, disease duration, and other comorbid diseases. Further analysis was carried out using logistic regression to confirm the above mentioned associations; the results assured that male gender and age under 60 years were the only factors significantly impacting improved glycemia (p<0.0001, OR=1.40, and p=0.03, OR=1.28, respectively).

**Table 2:** HbA1c scores throughout pre to post COVID-19 lockdown and subgrouping of the cohort based on the score changes beyond the clinically significant cut-off point (0.5%).

Outcome measure		Mean ( ± SD)	Mean difference
**HbA1c**	Pre-lockdown (%) Post-lockdown (%)	8.52 ( ± 1.5) 8.68 ( ± 1.6)	0.16 ( ± 0.1)
**Mean decreased by ≥0.5%**	n=1,064 (24.9%)	-1.38 ( ± 0.99)	-1.41 ( ± 75)*
**Mean increased by ≥0.5%**	n=1,574 (36.9%)	1.36 ( ± 0.85)	1.33 ( ± 61)*
**Mean changed by <0.5% (control group)**	n=1,628 (38.2%)	0.27 ( ± 0.24)	

*Mean difference in comparison to the control group.

**Table 3:** Association of different demographic variables with HbA1c changes by more or less than 0.5% throughout the lockdown period in T2DM patients.

Variables		HbA1c levels			p. value
		Changed by <0.5%	DECREASED by ≥0.5%	INCREASED by ≥0.5%	
**Gender**	Male (%)	648 (37.8%)	481 (28.1%)	585 (34.1%)	<0.0001
	Female (%)	980 (38.5%)	580 (22.8%)	984 (38.7%)	
**Nationality**	Saudi (%)	1,585 (38.1%)	1,034 (24.9%)	1,541 (37.0%)	0.23
	Non-Saudi (%)	43 (43.9%)	27 (27.6%)	28 (28.6%)	
**Regions**	Central (%)	1,321 (37.6%)	855 (24.3%)	1,336 (38.0%)	0.005
	Western (%)	179 (40.8%)	130 (29.6%)	130 (29.6%)	
	Eastern (%)	128 (41.7%)	76 (24.7%)	103 (33.6%)	
**Age (years)**	18-44 (%)	102 (35.1%)	82 (28.1%)	107 (36.8%)	<0.0001
	45-59 (%)	552 (35.2%)	439 (28.1%)	575 (36.7%)	
	≥60 (%)	974 (40.5%)	542 (22.5%)	889 (37.0%)	
**BMI**	Underweight (%)	5 (50.0%)	3 (30.0%)	2 (20.0%)	0.22
	Healthy (%)	108 (36.5%)	88 (29.7%)	100 (33.8%)	
	Overweight (%)	456 (37.9%)	315 (26.2%)	431 (35.9%)	
	Obese (%)	1,042 (38.4%)	645 (23.8%)	1,026 (37.8%)	
**Diabetes duration**	<5 years (%)	1,072 (38.0%)	720 (25.5%)	1,030 (36.5%)	0.45
	≥5 years (%)	556 (38.7%)	341 (23.7%)	539 (37.5%)	
Comorbidities					
Hypertension	Yes (%) No (%)	1,168 (38.7%) 460 (37.2%)	716 (23.7%) 345 (27.9%)	1,137 (37.6%) 432 (34.9%)	0.015
Hyperlipidemia	Yes (%) No (%)	1,490 (38.7%) 138 (34.0%)	941 (24.4%) 120 (29.5%)	1,421 (36.9%) 148 (36.5%)	0.05
Heart disease	Yes (%) No (%)	139 (40.1%) 1,489 (38.1%)	83 (23.9%) 978 (25.0%)	125 (36.0%) 1,444 (36.9%)	0.76
Kidney disease	Yes (%) No (%)	113 (33.1%) 1,515 (38.7%)	97 (28.4%) 964 (24.6%)	131 (38.5%) 1,438 (36.7%)	0.10

**Figure 2:**
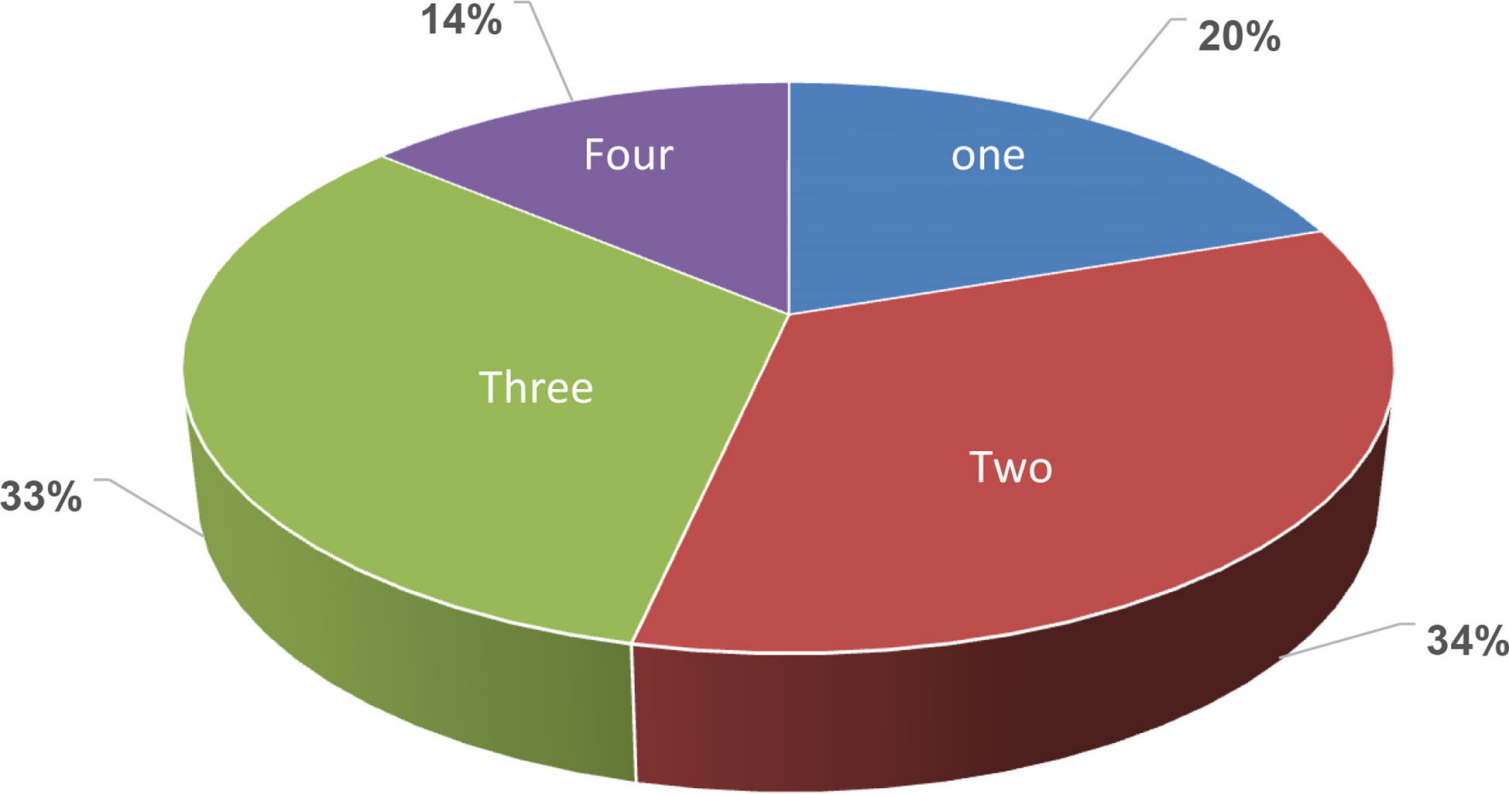
Percentage (%) of prescribed one or multiple anti-diabetic medications.

**Figure 3:**
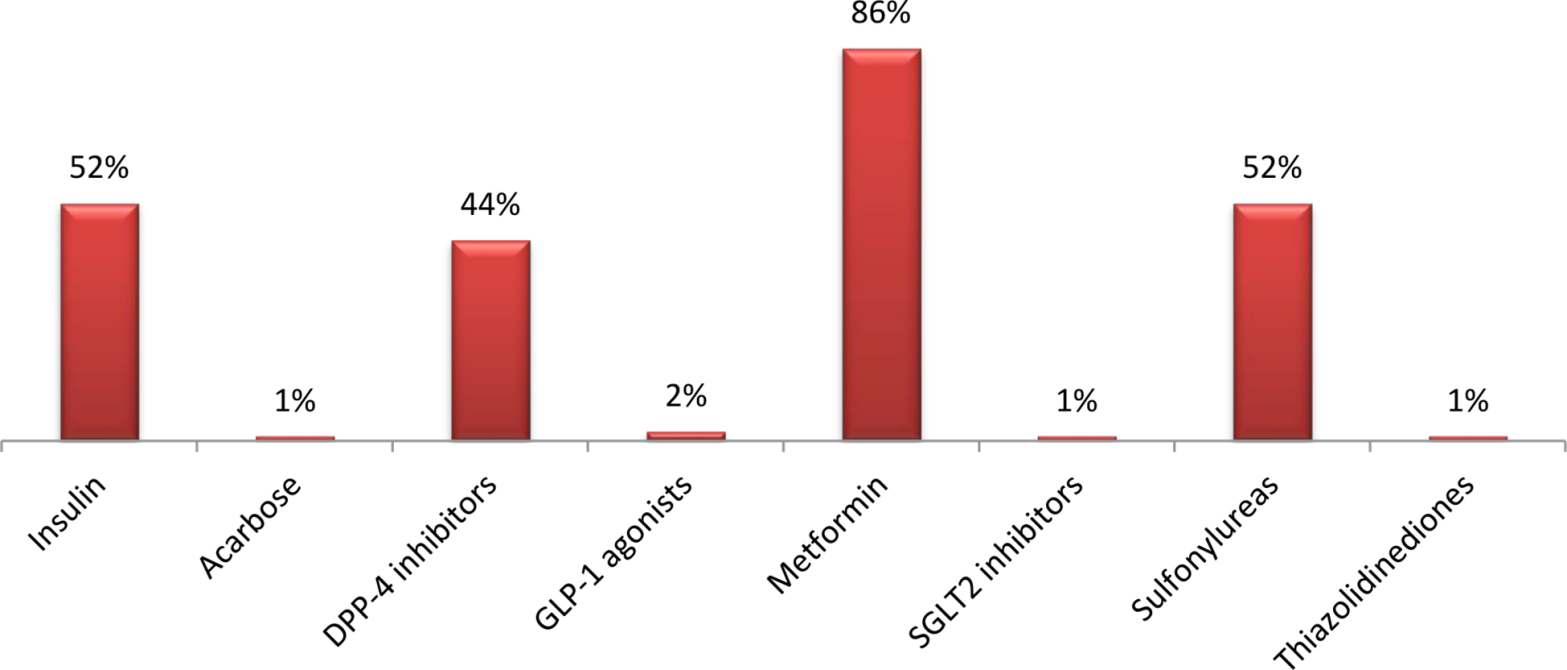
Percentage (%) of patients on different classes of anti-diabetic medications. Insulin: Regular, Aspart, NPH, Human insulin 70/30, Degludec, Detemir, Glargine. Alpha-glucosidase inhibitor: Acarbose. Dipeptidyl peptidase 4 (DDP-4) inhibitors: Linagliptin, Sitagliptin. Glucagon-like peptide-1 receptor (GLP-1) agonists: Liraglutide, Semaglutide. Biguanide: Metformin. Sodium-glucose cotransporter-2 (SGLT2) inhibitors: Dapagliflozin, Empagliflozin. Sulfonylureas: Glibenclamide, Gliclazide, Glimepiride, Glipizide. Thiazolidinedione: Pioglitazone.

We failed to access medication history of 348 (8.1%) patients, thus the analysis of the medication usage was restricted to 3,918 T2DM patients only. The results, shown in **Figure 2**, demonstrated that the majority of patients were using two (34%) or three (33%) medications. One fifth (20%) of the patients was using a single drug therapy, whereas 14% of the tested cohort were found to be on four drug-regimen. As shown in **Figure 3**, metformin represented the highest usage rate by the T2DM patients (86%), then sulfonylureas (glibenclamide, gliclazide, glimepiride, and glipizide) and insulin (both sulfonylureas and insulin groups were used by 55% of the patients). List of different types of used insulin are described in **Figure 3**. The dipeptidyl peptidase-4 (DPP-4) inhibitors (linagliptin or sitagliptin) were used by 44% of the patients. Other classes such as alpha-glucosidase inhibitor (acarbose), incretin mimetics (glucagon-like peptide-1 (GLP-1) agonists (liraglutide and semaglutide), sodium-glucose cotransporter-2 (SGLT2) inhibitors (dapagliflozin and empagliflozin), and thiazolidinedione (pioglitazone) were used by 1-2% of the patients. The comparison between the groups of patients who had a reduction and an increase in HbA1c by ≥0.5% revealed significant associations with use of insulin and sulfonylureas. **Table 4** shows that insulin users were lower in the group of decreased HbA1c levels than other patients on different oral antidiabetic agents (37.3% vs 43.8%, p=0.001, OR=0.76). Conversely, the individuals on oral sulfonylureas showed higher percentage in the reduced HbA1c group than patients on different treatment modalities (42.3% vs 37.9%, p=0.032, OR=1.20). Use of other major classes such as metformin and DDP-4 inhibitors, has no significant distribution differences among the compared cohorts. Likewise, no significant differences were noticed between patients on one or two medications in comparison to patients on three or four medications among the decreased group (40.3% vs 40.2%, p=1.0, OR=1.0).

**Table 4:** Impact of number of medications and their categories on directions of changes in HbA1c.

Medications	HbA1c levels DECREASED by ≥0.5% (n= 975)	HbA1c levels INCREASED by ≥0.5% (n= 1,445)	p-value	OR (95% CI*)
Number of medications				
1 or 2 (%)	499 (40.3%)	738 (59.7%)	1.0	1.0 (0.85-1.17)
3 or 4 (%)	476 (40.2%)	707 (59.8%)		
Insulin				
Yes (%)	492 (37.3%)	826 (62.7%)	0.001	0.76 (0.65-0.9)
No (%)	483 (43.8%)	619 (56.2%)		
Metformin				
Yes (%)	825 (39.6%)	1,259 (60.4%)	0.09	0.81 (0.64-1.03)
No (%)	150 (44.6%)	186 (55.4%)		
Sulfonylureas				
Yes (%)	550 (42.3%)	750 (57.7%)	0.032	1.20 (1.02-1.41)
No (%)	425 (37.9%)	695 (62.1%)		
DDP-4 inhibitors				
Yes (%)	453 (40.7%)	661 (59.3%)	0.76	1.03 (0.87-1.21)
No (%)	522 (41.3%)	784 (58.7%)		

CI*, Confidence Interval.

## Discussion

This study assessed the effectiveness of virtual clinics as an intervention for glycemic control in T2DM. In addition, impact of several demographic factors on individuals’ glycemic control was investigated. Our findings showed no significant change in mean HbA1c level (0.16% ± 0.1%) between pre and post lockdown period (March 2020 to June 2020). Whereas, significant improvements in glycemia were noticed more in males than females, in patients aged below 60 years, in non-hypertensive individuals, and in patients on sulfonylureas.

According to the American Diabetes Association and the American National Institute for Health and Clinical Excellence, the clinical significant cut-off point in HbA1c level is 0.5% (15, 16). The slight mean change observed in HbA1c level in our study before and after commencing the virtual clinics matches the finding reported in a meta-analysis of five randomized control trials that included 953 diabetic patients. The meta-analysis study concluded that telephone contact intervention was associated with a small and clinically insignificant change in HbA1c (mean HbA1c difference=-0.38%) (17). A recent Japanese study which involved 2,727 participants demonstrated that both telemedicine and clinic visit are effective in reducing mean HbA1c level in patients with pre- emergency levels of ≥7% only. Nevertheless, no differences were observed between both approaches (11). The slight increase detected in HbA1c value after lockdown in our study may partially related to the function and procedure of the newly introduced telemedicine approach and can be also explained by the decrease in patients’ physical activity and changes in dietary intake due to the increased time spent at home during lockdown. Thus, there is a drastic need to develop effective strategies to encourage diabetic patients to plan long-term lifestyle changes including healthy diet and regular exercise to improve their blood glucose levels in both normal conditions and in crises.

To ensure successfulness of telemedicine program, health care providers engaged in virtual clinics must be well-trained in professional communication with patients remotely (17–20). Recent research shows that the most common method of telemedicine intervention is *via* phone call not the video calling (17). Telephone is an effective communication model and is commonly used and accessed by almost everyone. Findings in Brazil suggest that phone calls are the simplest form of digital health for people with low literacy, such as the elderly (21). The current evidence supports the use of phone calls to provide simple information about the COVID-19 precautions (22–24). Telemedicine can be delivered through several other communication forms including online-based programs, videoconferencing, and sending text messages (18). Use of the new social media tools such as WhatsApp, SnapChat, and Telegram might be also beneficial. In comparison to our study which kept track of patients through phone calls only, the study conducted by Tourkmani et al. (2021) at Prince Sultan Military Medical City in Saudi Arabia used additional methods such as WhatsApp messenger to deliver written instructions, educational materials, and audio-visual aids. The patients were also asked to submit their self-monitoring blood glucose results over WhatsApp if they had any difficulty in submitting them during the virtual appointment. Providing this extra telehealth service may contributed in the noticed reduction of HbA1c level from 9.98% ± 1.33% pre-intervention to 8.32% ± 1.31% post-intervention (13). In another study which introduced a WeChat app as a remote tool for management of T2DM, the results showed higher treatment satisfaction and improved glycemic control (25).

Several demographic factors are thought to impact individuals’ glycemic control (26, 27). Obesity is highly prevalent in diabetic patients (28, 29), however, our study found no association between variability in HbA1c scores and different body mass index status in contrast to Kakade et al. (30). The data on the impact of gender on glycemic control is mixed. Our study found that males had higher frequent glycemic improvement than females, consistent with some but not all previous studies (31–34). Glycemic control may be worse in women due to differences in glucose hemostasis (35), psychological factors (36), and antidiabetic medication response (37). Unlike the findings of Benoit et al. (38) and Al-Lawati et al. (34), individuals below the age of 60 in our cohort showed a significant clinical decrease in HbA1c compared to older subjects, which is consistent with a previously published study (39). Furthermore, our results showed no impact of DM duration on patients response which is inconsistent with previous findings (34–43) which reported that the longer the duration course, the poorer glycemic outcome is predicted. In addition, our data does not support a previous study that involved 300 Saudi diabetic patients who showed positive correlation between reduced HbA1c and disease duration (44). Also, our results failed to confirm previous findings (30–45) in which patients with high lipid profile had poorer glycemic control.

The prevalence of hypertension among the selected cohort in our study was similar to a previous small study on diabetic patients (n=154), with 72% of whom were hypertensive (46). The study found no association between glycemic control levels and the concomitant blood pressure control status. In contrast, our data showed lower percentages of hypertensive patients who had clinical glycemic improvement in comparison to normotensive individuals. This was expected as previous reports emphasized that people with hypertension tend to have higher levels of insulin resistance than others (47). Similar to our findings, a study on Omani population found higher HbA1c levels among patients with elevated diastolic blood pressure (45). The prescription pattern of antidiabetic agents seen in our study is very similar to a previous Saudi study (48), where metformin, sulfonylureas, and insulin were the most common prescribed diabetic medications, the majority of patients were on a combination therapy, and no association detected between glycemic outcome and the number of used DM medications. However, our data showed that 44% of the patients were using DPP-4 inhibitors versus only 3.74% in the study conducted by Misbahuddin et al. (48). In our study, patients on insulin showed poorer glycemic control as reported previously (38), though other studies showed a positive impact of insulin use on HbA1c outcome (49, 50). A recent study revealed that use of insulin by T2DM patients may deteriorate their general health condition, induces psychological distress, and associated with activity difficulties in comparison to those using oral agents (51). On the other hand, our results indicated that the sulfonylureas were the only antidiabetic class which tends to significantly improve glycemic control.

Taken together, the implementation of telemedicine in managing many chronic diseases such as DM may take place in all healthcare system soon. This approach is useful to deliver health education and suggesting the necessary interventions to patients remotely. Nonetheless, telemedicine has some limitations such as inability to examine patients physically which is very likely needed when diagnosing new patients. Also, the patients may need to buy costly smart phones to be able to communicate with healthcare providers *via* social media tools. In addition, using unclear phone line or internet with low quality can halt the provided services (52, 53). Thus, developing a more comprehensive telemedicine approach and a more professional and well-trained team is predicted to effectively manage diabetic patients. Further prospective research is suggested to examine the cost effectiveness and impact of telemedicine on patients’ outcome in Saudi Arabia. Expansion of telemedicine presents an opportunity to generate easier solutions. Thus, the effects of telemedicine on hospital efficiency indicators and staff performance should be further studied.

## Conclusion

Our study illustrates that the virtual clinics scheduled during COVID-19 lockdown were effectively adopted at MNGHA to assist >63% of patients with T2DM maintain or lower HbA1c. More efforts are needed by health care providers and patients to achieve tighter glycemic controls. Telemedicine is a promising approach in managing patients with chronic diseases in particular diabetes. Thus, healthcare policymakers potentially need to consider use of telemedicine in normal times as well as in crises. Multidisciplinary health teams need to be carefully selected to promote practice of excellence and ensure the quality of care. More controlled prospective research is also needed to further evaluate the impact of telemedicine on T2DM. Variability in patients’ demographic and clinical characteristics such as gender, age, blood pressure status, use of insulin and sulfonylureas may play a role as determinants of glycemic outcome. More work is needed to determine the extent to which pre-diabetic patients (HbA1c 5.7 to 6.4%) developed diabetes during the COVID-19 lockdown.

## Data availability statement

The original contributions presented in the study are included in the article. Further inquiries can be directed to the corresponding authors.

## Ethics statement

The study, which involved human participants, was reviewed and approved by the Institutional Review Board (IRB) at King Abdullah International Medical Research Centre (reference number: NRC21R/463/11). Written informed consent for participation was not required for this study in accordance with the national legislation and the institutional requirements.

## Author contributions

AA and MA have conceived and conceptualized the work. SA analyzed the prescribed medications. AA, SA, and FA took part in the statistical analyses of data. KRB took the lead in reorganizing the manuscript and approving the final version. All authors shared in manuscript preparation. All authors have agreed to all of the submitted materials. All authors contributed to the article and approved the submitted version.
